# Socio-economic and agricultural factors associated with stunting of under 5-year children: findings from surveys in mountains, dry zone and delta regions of rural Myanmar (2016–2017)

**DOI:** 10.1017/S1368980023001076

**Published:** 2023-08

**Authors:** Min Kyaw Htet, Tran Thanh Do, Thet Wah, Thant Zin, Myat Pan Hmone, Shahreen Raihana, Elizabeth Kirkwood, Lwin Mar Hlaing, Michael J Dibley

**Affiliations:** 1 The University of Sydney, School of Public Health, Sydney, Australia; 2 National Institute of Nutrition, Hanoi, Vietnam; 3 NutriFirst, Yangon, Myanmar; 4 National Nutrition Centre, Ministry of Health and Sports, Nay Pyi Taw, Myanmar

**Keywords:** Food security, Stunting, Rural Myanmar, Under 5-year children

## Abstract

**Objective:**

The study’s objective was to investigate multiple underlying social, economic and agricultural determinants of stunting among under-five children in three distinct ecological areas in rural Myanmar.

**Design:**

Repeated cross-sectional surveys in three states of Myanmar.

**Setting:**

Rural households in Chin (mountainous), Magway (plains) and Ayeyarwady (delta).

**Participants:**

From two purposively selected adjacent townships in each state, we randomly selected twenty villages and, in each village, thirty households with under-five children. Households in the first survey in 2016 were revisited in late 2017 to capture seasonal variations.

**Results:**

Stunting increased from 40·4 % to 42·0 %, with the highest stunting prevalence in Chin state (62·4%). Univariate Poisson regression showed factors contributing to child stunting varied across the regions. Adjusted Poisson regression models showed that child’s age and short maternal stature (aRR = 1·14 for Chin, aRR = 1·89 for Magway and aRR = 1·86 for Ayeyarwady) were consistently associated with child stunting across three areas. For Chin, village-level indicators such as crop consumption (aRR = 1·18), crop diversity (aRR = 0·82) and land ownership (aRR = 0·89) were significantly associated with stunting. In Magway, the number of household members (aRR = 1·92), wealth status (aRR = 0·46), food security status (aRR = 1·14), land ownership (aRR = 0·85) and in Ayeyarwady, women’s decision-making (aRR = 0·67) and indicators related to hygiene (aRR = 1·13) and sanitation (aRR = 1·45) were associated with stunting.

**Conclusions:**

Area-specific factors were associated with stunting. Maternal short stature and child age were consistent determinants of stunting. A multi-sectoral local approach, including improvements in transport, is needed to address the intergenerational malnutrition problem.

Undernutrition among children widely exists in low- and middle-income countries, most of which are in Asia and Africa. Globally 21·3 % or 144 million children were reported stunted, and 24·7 % in Southeast Asia^(1)^. In Myanmar, formerly known as Burma, there is a challenge to understanding the magnitude of the undernutrition problem due to the lack of nationwide nutrition surveys. The first Demographic Health Survey in Myanmar was conducted in 2015–2016^(2)^, and the findings confirmed the existence of undernutrition among under-five children, with 29·2 % stunted and 57·8 % anaemic. These findings indicated stunting and anaemia as severe public health problems in Myanmar and needed attention^(3)^. Stunting reflects chronic undernutrition due to inadequate dietary intake and repeated bouts of infections. There is a link between the immediate and long-term consequences of child stunting and the underlying adverse environment, which also increases the risk of inadequate child growth and development^(4)^. A stunted child is less likely to achieve optimal brain development and higher education^(5)^. It was also possible that those children would miss their educational potential, consequently impacting the nation’s economy since each year of schooling was associated with increased wages by 7–11 %^(6)^.

Many factors contribute to child stunting, but household food security plays a fundamental role in child nutrition. Household food insecurity access score intends to measure the food security level in households, and it is frequently used for nutrition surveys^(7)^. Household food insecurity is a sensitive indicator reflecting socio-economic changes and, at the same time, influences the nutritional status of vulnerable groups in the household through its impact on dietary quality or access to nutrient-dense food^(8)^. Studies have shown that food insecurity is associated with the poor nutritional status of children. Children from moderately food-insecure households were 2·47 times more likely to be severely stunted (AOR = 2·47; 95 % CI (1·77, 3·46)), and those from severely food-insecure households were more likely to be severely stunted (AOR = 1·82; 95 % CI (1·34, 2·48)), compared with children aged 6–59 months from food-secure households^(9)^. A recently reported meta-analysis of twenty-one studies among 55 173 children and adolescents showed that food insecurity increased the risk of stunting OR: 1·17 (95 % CI: 1·09, 1·25), and the degree of food insecurity intensified the risk of stunting^(10)^. These studies underline the importance of food insecurity for child stunting but highlight that the link between food insecurity and undernutrition is complex. It is essential to have longitudinal data to understand these associations better. Dietary adequacy is an important factor for healthy child development and even in food-secure household, dietary adequacy for children is not guaranteed. Ensuring dietary adequacy requires good feeding practices, which can be assessed using UNICEF/WHO indicators for feeding practices and whenever possible, effort should be given to assess the feeding practice of children^(11)^. Myanmar has rich natural resources, agriculture is its main economic sector and it was once the main rice exporter in the region. Due to political instability, the country was left behind in all sectors, including health and nutrition. When the country initiated its democratic transition in 2010, there was a high expectation of improvements in the socio-economic status of the population and, eventually, the health and nutritional status of the people. However, it is unclear whether those residing in rural areas benefited from those socio-economic changes. We took this opportunity to investigate the impact of rapid economic transition on the food security and nutritional status of a vulnerable population in rural areas of the country in the early stage of a democratic transition. In this paper, we report the findings from two rounds of cross-sectional surveys (2016–2017) conducted in the same households in three geographical regions of the country.

The study’s objective was to investigate multiple underlying social, economic and agricultural determinants of stunting among under-five children in three distinct ecological areas in rural Myanmar.

## Methods

### Study design and setting

**Fig. 1:**
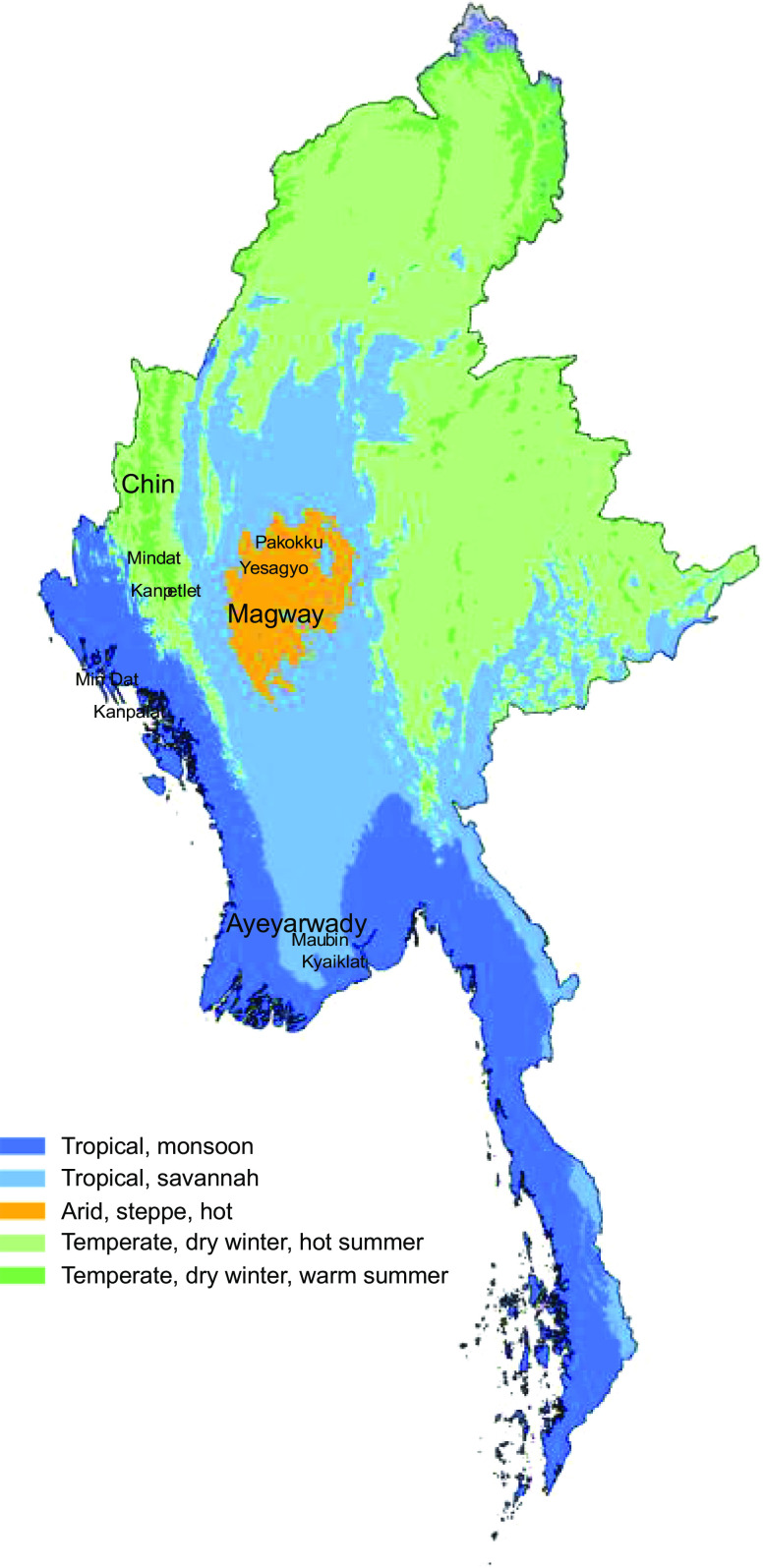
Locations of study townships

The study was a repeated cross-sectional panel survey conducted in early 2016 and late 2017 in rural Myanmar, covering pre- and post-harvest seasons. Field teams visited the same households in each round of the survey. We conducted the study in rural villages in three agro-economic zones of Myanmar: Chin state, a mountainous and food insecure area; Magway state, a central plain area with dry zone agricultural land and Ayeyarwady region, a river delta agricultural area (Fig. 1)^(12)^.

### Sampling methods and sample size

We purposively selected two adjacent townships in each zone, and from each township, we randomly selected twenty villages by probability proportionate to population size sampling. We randomly selected thirty households in each village to achieve 1200 households per area. A list of households from villages was obtained from township health offices, complemented by information from village midwives or primary health care workers. We listed the households with or without under-five children from each village before randomly selecting fifteen from each category. This process ensured we recruited a minimum number of under-five children from each village.

### Data collection

We developed a CommCare application to capture data using structured, error-detecting forms on Android tablets^(13)^. We recruited the survey teams from a local university (University of Community Medicine, Magway) and trained them to interview respondents using electronic data capture and measure anthropometry. Each survey collected data on background characteristics of households such as household socio-economic status, source of drinking water, types of toilets, women’s dietary diversity, household food insecurity, agricultural land access, home gardens, agricultural assets, types of crops, household assets and women’s decision making. In addition, we measured anthropometry for under-five children and women of reproductive age in the recruited households. The women in the household were most knowledgeable about the activities of household members and the food consumption pattern of families and were the survey respondents.

### Measures

Women’s decision-making: We classified women’s decision-making into three groups based on their responses to the six decision-making questions on (i) respondent’s health care, (ii) daily household purchase, (iii) large household purchase, (iv) visit to relatives, (v) types of food cook and (vi) using money. For each question, a score of three was given if the decision was made by ‘Respondent alone’, a score of one was given if the decision was made by ‘Respondent and husband/another male’ and a score of zero was given if the decision was made by ‘husband/another male alone’. Then, we calculated the weighted average score and categorised the scores ‘3’ as makes the decision by mother alone, ‘2’ as ‘makes the decision for at least three of the decisions alone and at least three of the decisions with her husband’, score ‘1’ as ‘at least six decisions together with her husband or a combination of fewer than six decisions with husband and less than four decisions alone’, and score ‘0’ as ‘no decision making’.^(14)^.

Household Food Insecurity: We assessed household food insecurity using the household food-insecurity access score to detect household food insecurity prevalence between the two surveys^(7)^. The module consists of nine sets of questions, each related to the occurrence and frequency of experiencing food insecurity in households. Based on the scores, the households were categorised into food secure, mildly food insecure, moderately food insecure or severely food insecure.

Women Dietary Diversity: We interviewed women from households about their consumption of different food groups in the last 24 h. The ten food groups in the survey followed FAO recommendations on minimum dietary diversity for women^(15)^. We defined minimum dietary diversity as women consuming five or more of the ten food groups.

Household wealth index: We constructed the household wealth index using the principal component analysis based on an inventory of the household’s ownership of consumer goods, household characteristics, source of drinking water, toilet facilities and other factors related to the household’s socio-economic status^(16,17)^. A total of twenty-eight variables were included in the index (sixteen for ownership of consumer goods, three for household characteristics, five for ownership of transport facilities, two for water source and treatment, cooking fuel and toilet facilities, respectively).

Village-level variables: We developed village-level scores to estimate the average village-level access to transportation, crop diversity and crop utilisation. We recoded each household transportation asset (bicycles, motorcycles, cars, light trucks or minibuses and boats) as one (for ownership) or zero (not owned). To construct the village transportation score, we summed these values for each household and then calculated the average of this score for each village. A higher score reflected more transportation assets and, therefore, more opportunities for household members to travel to local markets, district towns and health services.

Each survey recorded farm plot-level crops cultivated by households (with access to land) over the 12 months before the interview (excluding crops in home gardens) to estimate the crop species richness^(18)^. We recoded each crop as one (for cultivation in the last year) or zero (no cultivation). To construct the village crop diversity score, we summed these values for each farming household and then calculated the average of this score for each village. In total, we identified seventy-six different crop species or varieties (e.g. Emata rice - poor quality or Pawsan rice - better quality) cultivated in the survey areas.

We asked respondents about crop utilisation using a five-level scale, from keeping it all for their consumption (value 1) to selling it all (value 5). To construct the village crop utilisation score, we summed these values for each farming household and then calculated the average of this score for each village.

Anthropometric measurements: The same instruments were used for the anthropometric measurements of the mother and child. For weight measurement, we used SECA 874 digital scales (SECA GmbH & Co.KG, Hamburg, Germany) with accuracy to the nearest 0·1 g. we used ShorrBoards with precision to the nearest 0·1 cm (Weigh and Measure, LLC, Olney, Maryland, USA) for height. We took anthropometry measurements twice and a third if the difference between the first two measurements exceeded a predetermined allowable limit. The tablets were also programmed to warn when the recorded values were extreme to alert for a potential error and the need for re-measurement^(19)^. Length measurement was done in recumbent position for children under 2 years old and standing position for children above 2 years old. We conducted standardisation exercises for each anthropometrist before collecting the anthropometric data^(19)^. We determined the technical error of measurement and systematic error (bias) from the standardisation exercises, and if required, we gave them further training. We used the ‘2006 WHO Child Growth Standard’ to calculate child anthropometric Z-scores - height-for-age, weight-for-age and weight-for-length. We defined stunting, wasting, and underweight as less than –2·00 sd of height-for-age, weight-for-length and weight-for-age, respectively. Children with very low weight-for-height (Z score < –3sd) were referred to the local health department to assess and treat their severe acute malnutrition.

### Statistical analysis

We checked the normality of the distributions of continuous variables with the Kolmogorov–Smirnov test. We presented means (±sd) for normally distributed data and median with upper and lower quartiles for the not normally distributed data. We conducted univariate and multilevel mixed-effects Poisson regression analysis to identify the risk factors for stunting among under-five children. We calculated the variance inflation factor (VIF) value for each variable to check the multicollinearity among the independent variables. We included three random effects; one for the township strata, one for the village clusters and another for the repeated measurements of children nested within the clusters. We also used a robust Huber/White/sandwich estimator. We calculated sampling weights to adjust for oversampling of households with children under-five. The weights were based on sampling probabilities and were calculated separately for each cluster and applied in all analyses. We used STATA version 15 for all data analysis.

### Ethical consideration

This study was conducted according to the guidelines in the Declaration of Helsinki, and all procedures involving research study participants were approved by the Ethics Review Committee of the Department of Medical Research Myanmar (No.117/Ethics 2015) and the University of Sydney Human Research Ethics Committee. Written informed consent was obtained from all subjects/patients.

## Results

We recruited 3231 households in the first-round survey in early 2016 and revisited 90·4 % of the same households in late 2017 (*n* 2921). A total of 2049 under-five children participated in the first survey and 1696 in the second.

Table 1 shows the background characteristics of the children in each of the two surveys per region. Most mothers had achieved primary education, but most had some role in family decision-making. About 13 % of the mothers had short stature, and about 40 % of the households had more than five family members. Household wealth status remained similar in both survey rounds, but food-secure households increased from 19·6 % in the first to 28·2 % in the second round. The percentage of severely food insecure households decreased between the two survey rounds, from 16·4 % to 9·8 %. The households with women who achieved minimal dietary diversity score (DDS >= 5) tended to increase from 29·5 % to 40·0 % between the two survey rounds.

**Table 1:** Percent distribution of under-five children by region, survey rounds and selected background characteristics

	Chin						Magway						Ayeyarwady					
Characteristics	Round 1 (*n* 836)			Round 2 (*n* 742)			Round 1 (*n* 683)			Round 2 (*n* 546)			Round 1 (*n* 530)			Round 2 (*n* 408)		
		% CI	*n*		% CI	*n*		% CI	*n*		% CI	*n*		% CI	*n*		% CI	*n*
Age in year																		
< 12 months	12·8	10·4, 15·6	107	16·8	13·7, 20·5	125	13·9	11·3, 17·0	95	13	10·3, 16·3	71	11·9	8·9, 15·7	63	11·8	8·6, 15·9	48
12–23 months	22·8	20·1, 25·8	191	16·4	14·3, 18·8	122	23·6	21·1, 26·2	161	15·8	13·1, 18·8	86	19·6	16·7, 22·9	104	18·9	15·3, 23·0	77
24–35 months	22·5	19·7, 25·6	188	24·9	21·7, 28·5	185	21·5	18·3, 25·2	147	24·5	20·9, 28·6	134	20·4	16·6, 24·8	108	27	22·5, 31·9	110
36–47 months	23·9	21·5, 26·5	200	21·7	18·8, 24·9	161	20·6	17·8, 23·8	141	26·2	22·3, 30·5	143	20·4	17·2, 23·9	108	22·1	18·4, 26·2	90
48–59 months	17·9	15·8, 20·3	150	20·1	17·4, 23·0	149	20·4	18·1, 22·8	139	20·5	17·4, 24·0	112	27·7	24·3, 31·5	147	20·3	16·7, 24·6	83
Gender																		
Male	48·9	45·7, 52·2	409	51·2	47·8, 54·6	380	49·8	45·8, 53·8	340	50	45·4, 54·6	273	51·1	47·3, 55·0	271	55·4	50·8, 59·9	226
Female	51·1	47·8, 54·3	427	48·8	45·4, 52·2	362	50·2	46·2, 54·2	343	50	45·4, 54·6	273	48·9	45·0, 52·7	259	44·6	40·1, 49·2	182
Mother education																		
No formal	40·7	35·3, 46·3	340	58·4	53·8, 62·7	433	13·2	9·3, 18·3	90	34·6	29·9, 39·6	189	11·5	8·0, 16·3	61	32·6	27·6, 38·1	133
Primary	39·2	34·8, 43·9	328	26	23·0, 29·3	193	58·3	51·9, 64·3	398	44·7	38·9, 50·6	244	66·4	60·0, 72·3	352	51·5	45·5, 57·4	210
Middle and high	20·1	15·2, 26·0	168	15·6	11·6, 20·7	116	28·6	23·6, 34·1	195	20·7	16·7, 25·3	113	22·1	17·8, 27·0	117	15·9	12·1, 20·8	65
Women’s decision making*																		
Makes no decision	8·7	5·9, 12·8	73	7·5	4·7, 12·0	56	7·5	4·9, 11·3	51	8·5	6·2, 14·9	53	2·6	1·1, 6·3	14	3·0	1·5, 7·6	14
Makes decisions mostly with husband	63	55·8, 69·8	527	67·9	59·9, 75·1	504	49·8	45·3, 54·2	340	49·3	43·8, 53·6	266	42·3	35·4, 49·4	224	42·2	36·0, 48·6	172
Makes mostly her own decisions	28·2	22·2, 35·2	236	24·5	18·5, 31·8	182	42·8	38·3, 47·3	292	42·2	36·5, 46·8	227	55·1	48·2, 61·8	292	54·8	47·8, 60·9	222
Maternal height																		
Normal	77·4	72·3, 81·8	611	78·5	73·3, 82·9	372	92·3	89·7, 94·3	601	92·1	89·2, 94·2	348	93	89·6, 95·3	449	92·7	88·8, 95·3	255
Short stature (< 145 cm)	22·6	18·2, 27·7	178	21·5	17·1, 26·7	102	7·7	5·7, 10·3	50	7·9	5·8, 10·8	30	7·0	4·7, 10·4	34	7·3	4·7, 11·2	20
Household members																		
<= 5	39	33·9, 44·4	326	46·2	40·7, 51·9	343	68·2	64·5, 71·8	466	67·8	62·9, 72·3	370	64·9	59·9, 69·6	344	68·9	62·9, 74·3	281
> 5 members	61	55·6, 66·1	510	53·8	48·1, 59·3	399	31·8	28·2, 35·5	217	32·2	27·7, 37·1	176	35·1	30·4, 40·1	186	31·1	25·7, 37·1	127
Wealth index†																		
Lowest	40·3	33·0, 48·1	337	35·3	28·7, 42·6	262	6·7	5·0, 9·0	46	4·0	2·1, 7·5	22	30·4	26·1, 35·0	161	33·8	28·4, 39·7	138
Second	29·3	24·2, 35·0	245	30·1	26·0, 34·5	223	8·8	6·3, 12·2	60	7·3	4·6, 11·6	40	23	18·8, 27·9	122	24·5	19·6, 30·2	100
Third	14·6	11·0, 19·1	122	21·6	16·8, 27·3	160	22·7	19·3, 26·5	155	21·6	17·4, 26·5	118	20·4	17·1, 24·1	108	18·1	14·3, 22·7	74
Fourth	11·5	9·0, 14·5	96	9·6	6·4, 14·1	71	28	24·4, 31·8	191	27·5	23·5, 31·8	150	13·8	10·6, 17·7	73	15·2	11·6, 19·7	62
Highest	4·3	2·9, 6·3	36	3·5	2·2, 5·5	26	33·8	28·0, 40·2	231	39·6	32·4, 47·2	216	12·5	9·6, 15·9	66	8·3	5·4, 12·7	34
Household food security status‡																		
Food secure	6·5	4·3, 9·6	54	20·9	16·4, 26·2	155	32·7	27·5, 38·2	223	37·9	31·8, 44·4	207	23·6	18·7, 29·3	125	28·4	22·8, 34·9	116
Mild food insecure	14·1	10·5, 18·8	118	16·4	13·2, 20·2	122	20·5	17·4, 24·0	140	20·3	16·9, 24·2	111	13·4	10·0, 17·7	71	23	19·6, 26·9	94
Moderate food insecure	50·4	44·3, 56·4	421	48·5	41·9, 55·2	360	41·6	35·2, 48·2	284	37·5	32·1, 43·3	205	52·1	46·1, 58·0	276	39	33·7, 44·5	159
Severe food insecure	29·1	24·0, 34·7	243	14·2	10·5, 18·8	105	5·3	3·4, 8·1	36	4·2	2·5, 6·9	23	10·9	7·3, 16·0	58	9·6	6·0, 14·9	39
Women dietary diversity§																		
DDS < 5	82·8	78·1, 86·6	692	69·7	61·1, 77·1	517	51·5	46·1, 56·9	352	52·7	47·1, 58·3	288	75·5	70·6, 79·8	400	52·2	44·3, 60·0	213
DDS >= 5	17·2	13·4, 21·9	144	30·3	22·9, 38·9	225	48·5	43·1, 53·9	331	47·3	41·7, 52·9	258	24·5	20·2, 29·4	130	47·8	40·0, 55·7	195
Source of drinking water																		
Protected	29·2	21·6, 38·1	244	16·4	12·5, 21·3	122	85·7	72·2, 93·2	585	88·8	75·5, 95·4	485	32·3	24·0, 41·9	171	30·4	22·1, 40·2	124
Unprotected source	70·8	61·9, 78·4	592	83·6	78·7, 87·5	620	14·3	6·8, 27·8	98	11·2	4·6, 24·5	61	67·7	58·1, 76·0	359	69·6	59·8, 77·9	284
Sanitary latrine																		
Sanitary	89·8	82·5, 94·3	751	97·6	95·2, 98·8	724	76·4	68·7, 82·7	522	79·7	73·5, 84·7	435	94	89·9, 96·5	498	94·9	91·7, 96·8	387
Insanitary toilet	10·2	5·7, 17·5	85	2·4	1·2, 4·8	18	23·6	17·3, 31·3	161	20·3	15·3, 26·5	111	6·0	3·5, 10·1	32	5·1	3·2, 8·3	21
Presence of kitchen garden																		
Yes	31·7	25·6, 38·5	265	59·8	52·1, 67·1	444	16·7	11·9, 22·9	114	28·2	22·8, 34·3	154	16·4	12·4, 21·4	87	25·5	18·9, 33·4	104
No	68·3	61·5, 74·4	571	40·2	32·9, 47·9	298	83·3	77·1, 88·1	569	71·8	65·7, 77·2	392	83·6	78·6, 87·6	443	74·5	66·6, 81·1	304
Quartiles of village-average transportation score||																		
Q1 Least	73·6	55·4, 86·2	615	74·3	56·1, 86·7	551	7·5	2·3, 22·0	51	7·5	2·3, 22·2	41	15·1	6·2, 32·4	80	14·7	6·0, 31·7	60
Q2	26·4	13·8, 44·6	221	25·7	13·3, 43·9	191	33·5	19·6, 51·0	229	35·7	21·0, 53·7	195	39·2	25·0, 55·6	208	37·7	23·6, 54·3	154
Q3							59	42·1, 74·0		56·8	39·7, 72·4	310	38·7	23·8, 56·0	205	42·4	26·7, 59·8	173
Q4 Most													7·0	2·1, 20·8	37	5·1	1·4, 16·7	21
Village transport score																		
Mean	0·5			0·5			1·2			1·2			0·8			0·9		
sd	0·2			0·2			0·2			0·2			0·2			0·2		
Tertile of crop consumption score¶																		
Mostly consumed	92·9	74·6, 98·3	777	93·0	74·8, 98·3	690	17·6	8·5, 32·7	120	18·3	8·8, 34·3	100	3·0	0·4, 19·9	16	3·4	0·4, 22·1	14
Consumed some	7·1	1·7, 25·4	59	7·0	1·7, 25·2	52	29·0	16·3, 46·1	198	31·0	17·3, 48·9	169	58·1	41·0, 73·5	308	58·3	41·2, 73·7	238
Mostly sell							53·4	37·5, 68·7	365	50·7	34·7, 66·6	277	38·9	23·8, 56·4	206	38·2	23·4, 55·7	156
Village crop consumption score																		
Mean	2·4			2·4			4			4			4·1			4·1		
sd	0·7			0·7			0·5			0·5			0·3			0·3		
Tertile of village average crop diversity score**																		
Fewest crops	2·4	0·3, 16·4	20	1·2	0·2, 9·0	9	25·9	14·0, 42·8	172	26·7	14·1, 44·6	141	65·6	49·8, 78·5	343	66·7	51·3, 79·2	270
Medium	36·4	21·7, 54·1	304	35	20·6, 52·9	260	31·6	18·0, 49·4	210	31	17·3, 49·2	164	34·4	21·5, 50·2	180	33·3	20·8, 48·7	135
Most crops	61·2	43·6, 76·4	512	63·7	45·9, 78·5	473	42·5	27·3, 59·2	282	42·3	27·0, 59·4	224			0			0
Village crop diversity score																		
Mean	2·1			2·1			1·9			1·9			1·2			1·2		
sd	0·4			0·4			0·5			0·6			0·4			0·4		
Land ownership																		
None	22·6	16·7, 29·8	189	25·7	20·1, 32·3	191	66·5	58·5, 73·6	454	61·9	53·7, 69·5	338	72·8	66·7, 78·2	386	67·4	60·1, 74·0	275
Own land	77·4	70·2, 83·3	647	74·3	67·7, 79·9	551	33·5	26·4, 41·5	229	38·1	30·5, 46·3	208	27·2	21·8, 33·3	144	32·6	26·0, 39·9	133
Nutritional status of children																		
Stunting (HAZ < –2sd)	58·7	53·0, 64·3	491	62·4	57·4, 67·1	463	25·0	21·6, 28·8	171	22·7	18·8, 27·1	124	31·1	27·6, 34·9	165	30·6	26·8, 34·8	125
Underweight (WAZ < –2sd)	30·5	25·4, 36·2	255	27·8	23·9, 32·0	206	19·9	16·5, 23·9	136	24·2	20·5, 28·3	131	26·4	23·1, 30·0	140	27·1	23·0, 31·6	109
Wasting (WHZ < –2sd)	3·7	2·4, 5·8	31	2·4	1·5, 3·8	18	7·9	5·9, 10·6	54	7·0	4·8, 10·1	38	7·4	5·6, 9·7	39	9·0	6·7, 11·8	36

* Women’s decision-making was categorised into three groups (makes no decision, makes at least six decisions together with her husband or a combination of fewer than six decisions with husband and less than four decisions alone, makes the decision for at least three of the decisions alone and at least three of the decisions with her husband).

†Wealth index calculated by principal component analysis^(16)^.

‡Household food security was measured by household food security access score (HFIAS)^(7)^.

§Women’s dietary diversity was based on FAO^(15)^.

||Village transportation score estimates village-level access to transport based on household transportation assets.

¶Village crop consumption score was based on how the household used the crops.

** Village crop diversity score was based on the number of crops cultivated by households over the 12 months.

Overall, the prevalence of stunting increased from 40·4 % in the first round to 42·0 % in the second round. In the second survey, the highest stunting prevalence across survey rounds was in Chin State (62·4 %) (Table 2). The prevalence was low among under 1-year-old children but tended to increase by 2 years and older children. The prevalence of stunting among the children was lower if the mothers achieved higher education but tended to be higher if the mothers were short (< 145 cm). Children from higher wealth status and food-secure households tended to have a lower prevalence of stunting. At the village level, having better transportation was associated with a lower prevalence of stunting. On the other hand, children from households that consumed most of their crops had a higher prevalence of stunting.

**Table 2:** Percent distribution of under-five stunted children by region, survey rounds and selected background characteristics

	Chin						Magway						Ayeyarwady					
Stunting_1CAT	Round 1			Round 2			Round 1			Round 2			Round 1			Round 2		
		% CI	*n*		% CI	*n*		% CI	*n*		% CI	*n*		% CI	*n*		% CI	*n*
Age in year																		
< 12 months	28·0	19·2, 39·1	30	28·8	20·9, 38·3	36	11·6	6·4, 20·1	11	7·0	3·1, 15·1	5	11·1	5·6, 20·9	7	10·4	4·6, 21·9	5
12–23 months	56·5	48·9, 63·9	108	67·2	58·2, 75·1	82	22·4	16·3, 29·8	36	18·6	11·6, 28·6	16	26·9	19·1, 36·5	28	31·2	22·4, 41·5	24
24–35 months	59·6	51·8, 66·9	112	69·2	60·7, 76·6	128	35·4	27·4, 44·3	52	26·9	19·8, 35·4	36	40·7	32·1, 50·0	44	33·6	26·1, 42·1	37
36–47 months	69·5	60·8, 77·0	139	73·9	65·8, 80·7	119	26·2	19·1, 34·9	37	23·1	16·3, 31·7	33	30·6	23·0, 39·3	33	31·1	22·7, 40·9	28
48–59 months	68·0	57·3, 77·1	102	65·8	56·0, 74·4	98	25·2	17·8, 34·4	35	30·4	21·8, 40·5	34	36·1	29·0, 43·7	53	37·3	27·6, 48·3	31
Gender																		
Male	62·1	54·9, 68·8	254	65·8	58·4, 72·5	250	27·1	21·9, 33·0	92	24·2	19·3, 29·8	66	29·5	24·8, 34·8	80	29·6	24·0, 36·0	67
Female	55·5	49·4, 61·4	237	58·8	53·3, 64·2	213	23·0	18·9, 27·8	79	21·2	16·5, 26·9	58	32·8	27·2, 39·0	85	31·9	25·1, 39·4	58
Mother education																		
No formal	61·2	54·2, 67·7	208	57·5	51·1, 63·6	249	22·2	14·6, 32·4	20	18·5	12·9, 25·9	35	27·9	15·8, 44·4	17	19·5	13·8, 26·9	26
Primary	62·8	54·6, 70·3	206	75·6	68·8, 81·4	146	25·4	20·6, 30·8	101	25·8	19·8, 33·0	63	31·5	27·0, 36·4	111	36·7	31·1, 42·7	77
Middle and high	45·8	36·6, 55·3	77	58·6	49·0, 67·6	68	25·6	20·0, 32·2	50	23	15·6, 32·6	26	31·6	24·3, 40·0	37	33·8	24·2, 45·1	22
Women’s decision making*																		
Makes no decision	60·3	49·3, 70·3	44	62·5	53·7, 70·5	35	33·3	21·1, 48·4	17	22·6	12·0, 38·5	12	35·7	8·5, 76·9	5	14·3	4·3, 38·5	2
Makes decisions mostly with husband	58·3	51·3, 64·9	307	62·3	56·1, 68·1	314	23·5	19·6, 28·0	80	24·8	19·9, 30·5	66	35·3	29·2, 41·9	79	32	25·0, 39·9	55
Makes mostly her own decisions	59·3	51·4, 66·8	140	62·6	55·8, 69·0	114	25·3	20·5, 30·9	74	20·3	16·1, 25·2	46	27·7	23·5, 32·4	81	30·6	25·3, 36·5	68
Maternal height																		
Normal (> 145 cm)	57·3	50·8, 63·6	350	65·9	58·6, 72·5	245	24·5	20·8, 28·5	147	24·4	19·3, 30·3	85	28·7	25·0, 32·7	129	34·9	29·9, 40·3	89
Short stature (< 145 cm)	64·6	55·5, 72·8	115	80·4	72·0, 86·7	82	44·0	33·6, 54·9	22	50·0	31·4, 68·6	15	61·8	42·2, 78·2	21	55·0	31·6, 76·4	11
Household members																		
≤ 5 member	54·3	47·2, 61·2	177	58	51·4, 64·4	199	22·3	18·3, 26·9	104	18·6	14·4, 23·8	69	29·7	24·6, 35·3	102	30·2	25·1, 35·9	85
> 5 members	61·6	54·9, 67·8	314	66·2	60·8, 71·2	264	30·9	24·8, 37·7	67	31·3	24·4, 39·1	55	33·9	28·6, 39·5	63	31·5	23·6, 40·6	40
Wealth index†																		
Lowest	60·5	52·4, 68·1	204	63·4	54·6, 71·3	166	45·7	31·6, 60·5	21	36·4	21·0, 55·2	8	37·9	29·1, 47·6	61	29·0	22·3, 36·7	40
Second	58·4	50·1, 66·2	143	64·6	57·1, 71·4	144	23·3	14·2, 35·9	14	25	14·5, 39·6	10	32·8	25·7, 40·8	40	31·0	24·0, 39·0	31
Third	61·5	51·8, 70·3	75	61·9	53·0, 70·1	99	25·2	19·8, 31·5	39	19·5	10·4, 33·6	23	26·9	18·9, 36·6	29	43·2	32·8, 54·3	32
Fourth	55·2	44·6, 65·3	53	57·7	46·9, 67·9	41	24·6	18·3, 32·2	47	32·7	25·4, 40·9	49	31·5	22·3, 42·4	23	19·4	10·2, 33·5	12
Highest	44·4	27·0, 63·4	16	50·0	35·0, 65·0	13	21·6	16·7, 27·5	50	15·7	10·7, 22·5	34	18·2	9·7, 31·6	12	29·4	15·6, 48·4	10
Household food security status‡																		
Food secure	46·3	34·7, 58·3	25	67·7	58·0, 76·1	105	21·1	16·9, 25·9	47	21·7	16·0, 28·8	45	20·0	13·8, 28·0	25	25·0	18·5, 32·9	29
Mild food insecure	50	41·6, 58·4	59	57·4	46·3, 67·8	70	27·9	20·0, 37·4	39	18·9	11·8, 28·9	21	26·8	18·2, 37·6	19	29·8	21·7, 39·3	28
Moderate food insecure	62·5	55·4, 69·0	263	60·3	52·7, 67·3	217	26·1	21·0, 31·8	74	24·9	19·1, 31·7	51	35·5	30·6, 40·7	98	37·1	29·5, 45·4	59
Severe food insecure	59·3	51·8, 66·4	144	67·6	56·0, 77·4	71	30·6	15·8, 50·8	11	30·4	13·2, 55·8	7	39·7	28·3, 52·2	23	23·1	13·5, 36·5	9
Women dietary diversity§																		
Not achieved	59·8	53·7, 65·6	414	61·7	56·2, 66·9	319	26·1	21·8, 31·0	92	23·6	18·9, 29·1	68	34·8	30·6, 39·2	139	29·1	23·2, 35·9	62
Achieve MDD	53·5	44·1, 62·6	77	64·0	55·8, 71·4	144	23·9	19·2, 29·3	79	21·7	16·5, 28·1	56	20·0	14·0, 27·8	26	32·3	27·0, 38·2	63
Source of drinking water																		
Protected	64·8	57·6, 71·3	158	62·3	52·1, 71·5	76	24·8	21·0, 29·0	145	23·7	19·6, 28·3	115	29·2	22·0, 37·7	50	25	17·4, 34·6	31
Unprotected source	56·3	49·7, 62·6	333	62·4	56·9, 67·6	387	26·5	18·8, 36·1	26	14·8	7·5, 26·8	9	32·0	27·9, 36·4	115	33·1	28·7, 37·8	94
Sanitary latrine																		
Sanitary	58·2	52·1, 64·1	437	62·3	57·3, 67·0	451	23·9	20·5, 27·8	125	21·4	17·3, 26·1	93	30·1	26·6, 33·9	150	29·7	25·5, 34·3	115
Insanitary toilet	63·5	52·3, 73·4	54	66·7	38·8, 86·3	12	28·6	22·0, 36·3	46	27·9	18·7, 39·4	31	46·9	29·9, 64·6	15	47·6	28·7, 67·3	10
Presence of kitchen garden																		
Yes	52·8	43·0, 62·5	140	61·3	55·5, 66·7	272	28·9	21·9, 37·2	33	24·0	17·6, 32·0	37	27·6	20·2, 36·4	24	26·0	19·4, 33·9	27
No	61·5	56·1, 66·6	351	64·1	57·0, 70·6	191	24·3	20·5, 28·5	138	22·2	17·8, 27·4	87	31·8	27·6, 36·4	141	32·2	27·4, 37·4	98
Quartiles of village-average transportation score||																		
Q1 Least transportation	61·5	55·3, 67·3	378	64·6	59·2, 69·7	356							32·5	24·4, 41·8	26	31·7	28·6, 35·0	19
Q2	51·1	38·3, 63·8	113	56·0	44·7, 66·8	107	23·5	16·8, 32·0	12	19·5	12·9, 28·4	8	30·8	24·8, 37·5	64	33·1	25·4, 41·9	51
Q3							25·3	18·8, 33·1	58	23·6	15·9, 33·5	46	30·2	25·1, 36·0	62	27·7	22·9, 33·2	48
Q4 Most transportation							25·1	20·9, 29·8	101	22·6	18·2, 27·6	70	35·1	23·6, 48·7	13	33·3	28·8, 38·1	7
Village transport score																		
Mean	0·5			0·5			1·2			1·2			0·8			0·9		
sd	0·2			0·2			0·2			0·2			0·2			0·2		
Tertile of crop consumption score¶																		
Mostly consumed	57·9	52·0, 63·6	450	62·0	56·7, 67·0	428	30·0	23·4, 37·6	36	20·0	12·5, 30·4	20	31·3	31·3, 31·3	5	21·4	21·4, 21·4	3
Consumed some	69·5	49·0, 84·4	41	67·3	56·4, 76·6	35	29·8	23·2, 37·4	59	26·0	18·3, 35·6	44	28·6	24·0, 33·6	88	29·8	24·9, 35·2	71
Mostly sell							20·8	16·8, 25·5	76	21·7	16·9, 27·3	60	35·0	29·3, 41·1	72	32·7	26·5, 39·6	51
Village crop consumption score																		
Mean	2·4			2·4			4			4			4·1			4·1		
sd	0·7			0·7			0·5			0·5			0·3			0·3		
Tertile of village average crop diversity score**																		
Fewest no of crops	75·0	75·0, 75·0	15	88·9	88·9, 88·9	8	25·6	20·7, 31·2	44	24·8	19·0, 31·7	35	31·8	27·9, 35·9	109	30·4	25·4, 35·8	82
Medium of crops	55·3	44·4, 65·6	168	56·5	47·9, 64·8	147	23·3	17·0, 31·1	49	20·1	13·6, 28·7	33	30·6	23·7, 38·3	55	31·1	25·1, 37·8	42
Most no. of crops	60·2	53·2, 66·7	308	65·1	58·9, 70·8	308	27·0	22·0, 32·6	76	23·7	17·1, 31·8	53			0			0
Village crop diversity score																		
Mean	2·1			2·1			1·9			1·9			1·2			1·2		
sd	0·4			0·4			0·5			0·6			0·4			0·4		
Land ownership																		
None	63·5	53·9, 72·1	120	67·5	60·1, 74·2	129	24·7	20·5, 29·3	112	24·6	19·5, 30·5	83	33·7	29·1, 38·6	130	31·6	26·9, 36·8	87
Own land	57·3	50·8, 63·7	371	60·6	55·3, 65·7	334	25·8	20·7, 31·6	59	19·7	14·5, 26·3	41	24·3	18·9, 30·6	35	28·6	21·9, 36·3	38
Total	58·7	53·0, 64·3	491	62·4	57·4, 67·1	463	25·0	21·6, 28·8	171	22·7	18·8, 27·2	124	31·1	27·6, 34·9	165	30·6	26·7, 34·8	125

* Women’s decision-making was categorised into three groups (makes no decision, makes at least six decisions together with her husband or a combination of fewer than six decisions with husband and less than four decisions alone, makes the decision for at least three of the decisions alone and at least three of the decisions with her husband).

†Wealth index calculated by principal component analysis^(16)^.

‡Household food security was measured by household food security access score (HFIAS)^(7)^.

§Women’s dietary diversity was based on FAO^(15)^.

||Village transportation score estimates village-level access to transport based on household transportation assets.

¶Village crop consumption score was based on how the household used the crops.

** Village crop diversity score was based on the number of crops cultivated by households over the 12 months.

Univariate Poisson regression analysis showed that child age and maternal height were the consistent variables contributing to the risk of stunting among children in all three geographical areas (Table 3). In Chin State, maternal height (RR: 1·14 (95 % CI 1·1, 1·17)) the mother’s educational status (RR: 0·9 (95 % CI 0·86, 0·94) for middle school and higher) and agricultural-related variables such as crop consumption score (RR: 1·18 (95 % CI 1·18, 1·18)) for consuming most of the crops, crop diversity (RR: 0·75 (95 % CI 0·74, 0·77)) for having medium crops and (RR: 0·82 (95 % CI 0·68, 0·98) for having most number of crops) and land ownership (RR: 0·89 (95 % CI 0·86, 0·91)) were important factors associated with stunting in the adjusted model. In the adjusted model for Magway, maternal height (RR: 1·89 (95 % CI 1·81, 1·98)), the number of household members (RR: 1·92 (95 % CI 1·7, 2·1)), wealth status ((R: 0·46 (95 % CI 0·42, 0·49) for highest wealth status), food insecurity status (RR: 1·17 (95 % CI 1·14, 1·21) for mildly food insecure household) and land ownership (RR: 0·88 (95 % CI 0·83, 0·93)) were associated with stunting. Similarly, in Ayeyarwady, maternal height (RR: 1·86 (95 % CI 1·65, 2·09)), women’s decision making (RR: 0·67 (95 % CI 0·66, 0·69)), food insecurity status (RR: 1·13 (95 % CI 1·12, 1·14) for moderately food insecure households), hygiene, sanitation-related variables (RR: 1·13 (95 % CI 1·09, 1·17) for having unprotected drinking water and RR: 1·45 (95 % CI 1·29, 1·62) for having insanitary latrine) and crop consumption score (RR: 1·31 (95 % CI 1·02, 1·67)) were significantly associated with stunting in the adjusted model. The mean VIF value was 1·26, and there was no multicollinearity among the independent variables.

**Table 3:** Multilevel mixed-effects Poisson models to identify factors associated with child stunting in three regions in Myanmar

Variable	Chin						Magway						Ayeyarwady					
	RR univariate*	95 % CI	*P*-value	RR adjusted†	95 % CI	*P*-value	RR univariate	95 % CI	*P*-value	RR adjusted	95 % CI	*P*-value	RR univariate	95 % CI	*P*-value	RR adjusted	95 % CI	*P*-value
Age of child																		
< 12 months	Ref			Ref			Ref			Ref			Ref			Ref		
12–23 months	2·07	1·57, 2·73	<0·001	1·86	1·23, 2·81	<0·001	2·4	1·5, 3·84	<0·001	1·66	0·81, 3·39	0·17	3·75	1·32, 10·62	0·01	3·91	1·07, 14·27	0·04
24–35 months	2·21	1·55, 3·14	<0·001	1·96	1·2, 3·2	0·01	3·84	2·44, 6·04	<0·001	2·76	1·58, 4·8	<0·001	4·77	2·43, 9·36	<0·001	4·6	3·19, 6·63	<0·001
36–47 months	2·44	1·88, 3·18	<0·001	2·25	1·46, 3·46	<0·001	2·5	1·46, 4·28	<0·001	1·82	0·79, 4·16	0·16	3·86	1·92, 7·78	<0·001	3·75	2·29, 6·15	<0·001
48–59 months	2·29	1·57, 3·34	<0·001	2·03	1·21, 3·4	0·01	3·44	2·5, 4·73	<0·001	2·55	1·49, 4·36	<0·001	4·25	1·83, 9·84	<0·001	4·26	2·41, 7·55	<0·001
Gender																		
Female	0·89	0·78, 1·03	0·11	0·91	0·78, 1·05	0·21	0·84	0·58, 1·22	0·36				1·18	1, 1·3	0·02	1·3	0·93, 1·8	0·12
Mother education																		
No formal	Ref			Ref			Ref			Ref			Ref			Ref		
Primary	1·16	1·14, 1·17	<0·001	1·07	1·01, 1·13	0·02	1·23	1·15, 1·32	<0·001	1·05	0·77, 1·44	0·74	1·6	1·01, 2·53	0·05	1·29	0·38, 4·39	0·68
Middle and high	0·91	0·89, 0·92	<0·001	0·9	0·86, 0·94	<0·001	1·16	0·95, 1·4	0·14	1·12	1, 1·25	0·05	1·53	0·75, 3·1	0·24	1·31	0·28, 6·03	0·73
Women’s decision making‡																		
Makes no decision	Ref			Ref			Ref			Ref			Ref			Ref		
Makes decisions mostly with husband	1·01	0·78, 1·3	0·93				1·04	0·82, 1·33	0·75				0·97	0·72, 1·31	0·85	0·68	0·68, 0·69	<0·001
Makes mostly her own decisions	1·04	0·67, 1·62	0·87				0·94	0·66, 1·34	0·75				0·78	0·66, 0·93	<0·001	0·67	0·66, 0·69	<0·001
Maternal height																		
Short stature (<145 cm)	1·15	1·05, 1·27	<0·001	1·14	1·1, 1·17	<0·001	1·81	1·8, 1·83	<0·001	1·89	1·81, 1·98	<0·001	1·89	1·6, 2·3	<0·001	1·86	1·65, 2·09	<0·001
Household members																		
Members >= 5	1·11	0·94, 1·31	0·20	1·06	0·92, 1·24	0·41	1·79	1·55, 2·06	<0·001	1·92	1·7, 2·16	<0·001	1·05	0·76, 1·45	0·75			
Wealth index§																		
Lowest	Ref			Ref			Ref			Ref			Ref			Ref		
Second	0·97	0·71, 1·34	0·87				0·46	0·34, 0·62	<0·001	0·46	0·44, 0·49	<0·001	0·92	0·91, 0·93	<0·001	0·98	0·9, 1·06	0·56
Third	0·95	0·72, 1·26	0·74				0·47	0·3, 0·73	<0·001	0·42	0·29, 0·6	<0·001	1·08	0·88, 1·32	0·47	1·25	0·9, 1·73	0·19
Fourth	0·85	0·55, 1·3	0·45				0·64	0·53, 0·78	<0·001	0·65	0·62, 0·68	<0·001	0·76	0·47, 1·24	0·28	0·96	0·53, 1·73	0·88
Highest	0·71	0·38, 1·31	0·27				0·42	0·41, 0·43	<0·001	0·46	0·42, 0·49	<0·001	0·62	0·37, 1·02	0·06	0·66	0·34, 1·28	0·22
Food security status||																		
Food secure	Ref			Ref			Ref			Ref			Ref			Ref		
Mildly Insecure	0·92	0·74, 1·15	0·48				1·25	1·22, 1·29	<0·001	1·17	1·14, 1·21	<0·001	1·15	1·11, 1·19	<0·001	0·99	0·86, 1·15	0·89
Moderately Insecure	0·99	0·76, 1·29	0·94				1·18	0·97, 1·44	0·09	0·99	0·81, 1·22	0·96	1·39	1·26, 1·53	<0·001	1·13	1·12, 1·14	<0·001
Severely Insecure	1	0·68, 1·46	1·00				1·33	1·28, 1·38	<0·001	1·14	0·99, 1·3	0·06	1·35	1·13, 1·62	<0·001	1·15	0·93, 1·43	0·20
Dietary diversity^¶^																		
W-DDS > 5	0·99	0·78, 1·27	0·96				0·81	0·64, 1·02	0·07	0·9	0·71, 1·15	0·39	0·84	0·6, 1·1	0·28			
Water source																		
Unprotected	0·91	0·79, 1·06	0·22	0·91	0·74, 1·12	0·37	0·99	0·67, 1·47	0·97				1·22	1·2, 1·3	<0·001	1·13	1·09, 1·17	<0·001
Toilet																		
Insanitary	1·03	0·85, 1·24	0·79				1·28	1·09, 1·5	<0·001	1·06	0·74, 1·51	0·76	1·71	1·5, 2	<0·001	1·45	1·29, 1·62	<0·001
Kitchen garden																		
Yes	0·9	0·8, 1	0·34				0·9	0·5, 1·7	0·91				0·8	0·4, 1·5	0·55			
Transportation score**																		
Village-level transport score	0·6	0·57, 0·62	<0·001	0·9	0·81, 1·02	0·09	1·19	0·54, 2·61	0·66				1·01	0·69, 1·48	0·96			
Village Crop consumption††																		
Village crop consumption score	1·21	1·11, 1·33	<0·001	1·18	1·18, 1·18	<0·001	0·93	0·72, 1·19	0·55				1·3	0·9, 1·8	0·10	1·31	1·02, 1·67	0·03
Village Crop diversity‡‡																		
Few	Ref			Ref			Ref			Ref			Ref			Ref		
Medium number of crops	0·72	0·66, 0·78	<0·001	0·75	0·74, 0·77	<0·001	0·89	0·74, 1·07	0·21	0·84	0·53, 1·35	0·48	0·89	0·72, 1·12	0·33			
Most number of crops	0·82	0·72, 0·92	<0·001	0·82	0·68, 0·98	0·03	1·04	0·8, 1·37	0·75	1·09	0·86, 1·37	0·47						
Land ownership																		
Own land	0·89	0·84, 0·94	<0·001	0·89	0·86, 0·91	<0·001	0·85	0·81, 0·89	<0·001	0·88	0·83, 0·93	<0·001	0·77	0·6, 1	0·07	0·86	0·71, 1·02	0·09
Survey round																		
Second round	1·05	1·03, 1·07	<0·001	1·08	0·98, 1·19	0·13	0·94	0·83, 1·07	0·36				1	0·92, 1·08	0·95	0·02	0·02, 0·03	<0·001

* Relative risk for stunting by univariate multilevel mixed-effect Poisson regression analysis, significant at *P* < 0·05.

†Relative risk for stunting by multilevel mixed-effect Poisson regression analysis, adjusted for covariables, significant at *P* < 0·05.

‡Women decision making was categorised into three groups (makes no decision, makes at least six decisions together with her husband or a combination of fewer than six decisions with husband and less than four decisions alone, makes the decision for at least three of the decisions alone and at least three of the decisions with her husband)^(14)^.

§Wealth index calculated by principal component analysis from an inventory of household assets and facilities^(16)^.

||Household food security was measured by household food security access score (HFIAS)^(7)^.

^¶^Women dietary diversity used recall of at least five out of ten food groups as recommended by FAO^(15)^.

** Village transportation score estimates village-level access to transport based on household transportation assets.

††Village crop consumption score estimates the household’s use of the crops with higher values indicating more selling than consumption of crops.

‡‡Village crop diversity score was based on the number of crops cultivated by households over the 12 months before the interview.

## Discussion

This unique panel study followed the same households in two rounds of surveys in rural areas from three agro-climate regions, hilly, central dry zone and delta regions of Myanmar. Among the three regions, we found the highest prevalence of stunting in Chin State, and it increased between the two survey rounds. However, the prevalence was lower in both Magway and Ayeyarwady regions. Factors contributing to the stunting varied across the three regions, but we identified the child’s age and maternal height as consistently associated with child stunting in all regions. In Chin State, village-level variables were important factors related to child stunting; in Magway, it was wealth status, land ownership and number of household members; while in Ayeyarwady, it was women’s decision-making and indicators related to hygiene and sanitation. Food security status improved in all regions between the two rounds of surveys, and in Ayeyarwady and Magway, household food security was an important factor contributing to stunting.

The strength of this study was the geographic coverage since it covered rural areas in three different geographical regions, including hard-to-reach areas in both surveys. The teams successfully followed up with more than 90 % of the households. The panel dataset generated from the study allowed us to compare the food security status, food consumption patterns and nutritional status of under-five children over 2 years in the same households over time. The study identified common factors related to stunting in all three areas and region-specific factors, which are important for planning and implementing nutrition programs in the country. The limitation of the study was that the dietary intake or feeding practices of child, which is the immediate determinants of child stunting, were not collected in both rounds of surveys and thus we could not include these important factors in the analysis models. Distribution of household expenditure was also not collected in the surveys, which could provide a better understating of household-level determinants of stunting. Another limitation was that we could not validate the transportation index in the current study. Nonetheless, we included the variable in the analysis since it was an important factor for identifying the risk of having limited access to food and markets.

The findings from our surveys were consistent with previous national surveys. The Myanmar DHS (2015–2016) reported that the prevalence of stunting among under-five children in Chin state was 41·0 %, the highest in the country, while it was 37·2 % in Ayeyarwady and 25·9 % in Magway^(2)^. Similarly, a nationwide micronutrient survey (Myanmar Micronutrient and Food Consumption Survey MMFCS) conducted in 2017–2018 reported the prevalence of stunting in Chin was 40 %, followed by 37·9 % in Ayeyarwady and 22·4 % in Magway^(20)^. These findings are consistent with our survey results. We also found the highest prevalence of stunting in Chin state, followed by Ayeyarwady and Magway.

However, our results differed from an earlier analysis^(21)^ of the same data used in this study. They reported a negative association between greater diversity of household agricultural production and child nutrition. In contrast, we found a protective effect of crop diversity for stunting in Chin but no effect in other states. We observed higher crop diversity in Chin State, which has the highest child stunting levels. The earlier analysis pooled the data from the three states and may have been confounded by the state of residence. A further difference between our results and the earlier analysis is for kitchen gardens. We found no association between the presence in the household of a kitchen garden and child stunting. In contrast, Vu and Rammohan (2021) reported a protective association^(21)^.

The child’s age was consistently associated with stunting in all study regions, the older the child’s age, the higher the stunting rate. A study in Nepal reported similar findings with child age as one of the significant determinants of stunting, even after adjusting for family size, household headship and household food security status^(22)^. The most common reason is poor child feeding practices, which leads to inadequate intake of nutrients for the full growth potential of the children, especially through sub-optimal complementary feeding practices. There is a need for counselling on appropriate infant and young child feeding from the start of ante-natal care.

Our finding also showed that maternal height was a consistent determinant of stunting for children among the three regions. This finding demonstrated the widespread existence of an intergenerational problem of undernutrition in the country’s rural areas in all three different agro-ecological zones. To date, not much is known about the interplay between genetics, epigenetics and environmental determinants of stunting. But studies show a strong association between maternal stature and child stunting^(23–25)^ and suggest it is a strong and reliable maker for assessing intergenerational growth faltering. The finding from a pooled analysis of fifty-four low- to middle-income countries reported that the risk of stunting among offspring of short-statured mothers (< 145 cm) was more than two times higher (RR = 2·132, 95 % CI: 2·103, 2·161) compared with tall mothers^(26)^. A study from Bangladesh using the dataset of four rounds of DHS surveys reported the risk of stunting was more than two times higher (OR: 2·10, 95 % CI: 1·97, 2·23) among children of the short-statured mothers and confirmed the existence of intergenerational undernutrition problem in the country^(27)^. Similarly, a recent paper from Indonesia also indicted that children from mothers and fathers with short stature had almost six times likelihoods of being stunted at birth^(28)^.

The study’s primary purpose was to investigate the livelihoods and food security status of households in rural areas of the country during the rapid social-economic transition since the country experienced its democratic period for the first time. Overall, our findings showed that food security improved between the two survey rounds. The number of food-secure households increased from 19·6 % at the first round to 28·2 % at the second round, with more food-secure households in Magway. The adjusted regression analysis showed household food insecurity was an important determinant of stunting for children in Ayeyarwady and Magway, but not in Chin. Ayeyarwady was the most agriculturally productive region in the country, and normally, rice produced from this region is distributed to other parts of the country. However, after the devastating cyclone Nargis in 2008, there was a major impact on the population’s livelihoods, and people in rural areas are still vulnerable^(29)^. Our findings highlighted that the people in the area have still not fully recovered from the consequences of the cyclone and that food security remains a major issue in the area, contributing to an increased risk of stunting among children.

Magway is in the central dry zone of the country, typically characterised by having lower rainfall compared to the rest of the country. We found the number of household members and household wealth status was significantly associated with child stunting in the region. A study in Cambodia has similarly reported the number of household members as the risk factor of stunting. In addition, a pooled analysis of three Cambodian demographic health surveys identified maternal height, child age and gender, parents’ education and household wealth status as the risk factors for stunting^(24)^. Similarly, in Indonesia, a study reported children from households with five to seven members had a higher risk for stunting^(25)^. These results suggested that children were most vulnerable, especially among households with large family sizes. In addition, our findings showed that household wealth status and land ownership played a significant role in child stunting. This finding was consistent with earlier reports of land and livestock ownership as major determinants of household food security in the dry zone and the association between the ownership of land and livestock and maternal BMI in this arid zone^(30)^. On the other hand, most of the population in the area does not own land, according to a JICA report^(31)^.

Chin is the hilly region of the country, and most of the population lives in hard-to-reach areas and mainly relies on their self-production of agricultural produce. Access to health care or education is challenging, and as a result, the region is considered one of the least developed areas in the country. It is also prone to rodent infestation, and with each rodent infestation, people suffer from more severe food insecurity, with such events contributing to secular trends in undernutrition. Although our findings showed that food security status was not a significant determinant of child stunting in Chin State, the prevalence of food-insecure households was the highest in Chin State in both rounds of surveys 93·5 % and 79·1 %, respectively. Earlier surveys conducted in 2011–2012 in the same townships (Min Dat and Kan Pat Let) showed more than 96 % of the household experienced food shortages in the past 12 months^(32)^.

The finding from MMFCS showed Chin and Magway were among the states with the highest rate of food-insecure households, but most households in Ayeyarwady were relatively food secure (77·5 %). In contrast, we found more food-secure households in the Magway region among the three states in both survey rounds. One of the possible reasons was the difference in the sampling of households in MMFCS, which covered a wider population, while in our surveys, the sampling was limited to two purposively selected townships in each region. In Chin State, village-level indicators such as crop consumption score and crop diversity score were significant determinants for stunting of children. In a normal year, farmers in Chin are not self-sufficient, and they are not well equipped with technology and resources to cope with increased erosion and decreased soil fertility^(32)^. Earlier studies reported access to markets had a stronger relationship with dietary diversity compared to production diversity^(33)^. Better transportation and communication would further improve the livelihoods and eventually the population’s nutritional status.

Mothers are the primary person responsible for preparing nutritious food in the family and play an important role in the nutritional status of children in the family. But in the current study, women’s dietary diversity showed no significant contribution to child stunting, a finding that requires further investigation. A survey conducted among Bangladesh women reported that 58 % of mothers of stunted children did not achieve minimum dietary diversity for women (< 5 groups), and the risk of child stunting was 1·7 times higher compared to those mothers who achieved minimum dietary diversity for women^(34)^. The finding suggests that achieving minimum dietary diversity among women is an important factor contributing to child stunting, and it needs more attention in programs. A study from northern Thailand reported widespread food insecurity and that stunted children were less likely to meet minimum dietary diversity^(35)^. A study among 15–19 years young women working in the garment sector in Yangon, Myanmar, showed that food security level was one of the risk factors for achieving minimal women dietary diversity score among these women^(36)^. Our study showed an improvement in women’s dietary diversity in Chin and Ayeyarwardy but not in Magway between the two survey rounds. It is likely that the women’s dietary diversity increased with improved socio-economic conditions, but the lack of improvement in women’s dietary diversity in Magway needs further exploration.

Women’s leadership or decision-making in the family plays an important role in achieving household food security. Our findings showed that most women who participated in the survey hold at least some role in the decision-making process in the family. Using two rounds of DHS surveys, a report from Malawi suggested that maternal autonomy was associated with a lower risk of early childhood stunting^(37)^. In our survey, women’s decision-making in the households did not appear as an important contributor to child stunting in Chin and Magway, but interestingly, it was in Ayeyarwady. Background education status of women was comparable across the three states, and distribution of household expenditure could further explain the discrepancies. Findings from Indonesia suggest an association between smoking and food insecurity among poor households as smoking uses some of the household expenses that could be used for food^(38)^. However, this information on household expenditure was unavailable in our study, but it needs further exploration in future studies.

The findings showed a long-standing problem with undernutrition among the rural population, and more comprehensive interventions are required to address maternal and child nutritional problems. Since agriculture is the major source of income generation for the population, improving the agricultural sector is essential to improve the livelihoods and nutritional status of the population in rural areas. Following the democratic transition in 2010, with the support from the international trust fund, a multi-sectoral approach for improving the population’s nutritional status was developed known as the Multi-sectoral National Plan of Action on Nutrition (MS-NPAN)^(39)^. Understanding the importance of the links between different sectors in nutrition, the Ministry of Health and Sports, together with four other ministers (Ministry of Education, Ministry of Agriculture, and Ministry of Social Welfare), endorsed the MS-NPAN. This agreement was encouraging for the country and an important step toward improving the population’s nutritional status since it provided a strong foundation for tackling undernutrition.

As a consequence of the global COVID-19 pandemic, the impact on food security was inevitable worldwide, especially among vulnerable populations due to disruptions of the supply chain and production. In addition, Myanmar people faced another political turmoil magnifying the underlying existence of food security problems amid COVID-19. World Food Program estimates about four million people in Myanmar will face food insecurity in 2022^(40)^. There is an imminent negative impact on decades of hard work and resources to improve livelihoods, food security and child stunting. Policymakers should consider approaches to improve the determinants of stunting, such as improving food security, dietary diversity, women empowerment when implementing National Nutrition programmes and strategies like MS-NPAN, which will tackle the multi-dimensional nature of undernutrition. It may take time to develop a new comprehensive nutrition programme such as MS-NPAN. Nonetheless, in the future, it should consider innovative approaches to breaking the problem of intergenerational undernutrition based on our findings.
