# Socio-economic and agricultural factors associated with stunting of under 5-year children: findings from surveys in mountains, dry zone and delta regions of rural Myanmar (2016–2017) – CORRIGENDUM

**DOI:** 10.1017/S1368980023001404

**Published:** 2023-09

**Authors:** Min Kyaw Htet, Tran Thanh Do, Thet Wah, Thant Zin, Myat Pan Hmone, Shahreen Raihana, Elizabeth Kirkwood, Lwin Mar Hlaing, Michael J Dibley

A previous version of this article listed the wrong affiliation for co-author Shahreen Raihana. The article has since been updated to list the correction affiliation.
